# Deep learning-based classification of breast cancer cells using transmembrane receptor dynamics

**DOI:** 10.1093/bioinformatics/btab581

**Published:** 2021-08-15

**Authors:** Mirae Kim, Soonwoo Hong, Thomas E Yankeelov, Hsin-Chih Yeh, Yen-Liang Liu

**Affiliations:** Department of Computer Science, Rice University, Houston, TX 77005, USA; Department of Biomedical Engineering, The University of Texas at Austin, TX 78712, USA; Department of Biomedical Engineering, The University of Texas at Austin, TX 78712, USA; Oden Institute for Computational Engineering and Science, The University of Texas at Austin, TX 78712, USA; Department of Diagnostic Medicine, The University of Texas at Austin, TX 78712, USA; Department of Oncology, The University of Texas at Austin, TX 78712, USA; Livestrong Cancer Institutes, The University of Texas at Austin, TX 78712, USA; Department of Biomedical Engineering, The University of Texas at Austin, TX 78712, USA; Texas Materials Institute, The University of Texas at Austin, TX 78712, USA; Master Program for Biomedical Engineering, China Medical University, Taichung 40678, Taiwan; Graduate Institute of Biomedical Sciences, China Medical University, Taichung 40678, Taiwan

## Abstract

**Motivation:**

Motions of transmembrane receptors on cancer cell surfaces can reveal biophysical features of the cancer cells, thus providing a method for characterizing cancer cell phenotypes. While conventional analysis of receptor motions in the cell membrane mostly relies on the mean-squared displacement plots, much information is lost when producing these plots from the trajectories. Here we employ deep learning to classify breast cancer cell types based on the trajectories of epidermal growth factor receptor (EGFR). Our model is an artificial neural network trained on the EGFR motions acquired from six breast cancer cell lines of varying invasiveness and receptor status: MCF7 (hormone receptor positive), BT474 (HER2-positive), SKBR3 (HER2-positive), MDA-MB-468 (triple negative, TN), MDA-MB-231 (TN) and BT549 (TN).

**Results:**

The model successfully classified the trajectories within individual cell lines with 83% accuracy and predicted receptor status with 85% accuracy. To further validate the method, epithelial–mesenchymal transition (EMT) was induced in benign MCF10A cells, noninvasive MCF7 cancer cells and highly invasive MDA-MB-231 cancer cells, and EGFR trajectories from these cells were tested. As expected, after EMT induction, both MCF10A and MCF7 cells showed higher rates of classification as TN cells, but not the MDA-MB-231 cells. Whereas deep learning-based cancer cell classifications are primarily based on the optical transmission images of cell morphology and the fluorescence images of cell organelles or cytoskeletal structures, here we demonstrated an alternative way to classify cancer cells using a dynamic, biophysical feature that is readily accessible.

**Availability and implementation:**

A python implementation of deep learning-based classification can be found at https://github.com/soonwoohong/Deep-learning-for-EGFR-trajectory-classification.

**Supplementary information:**

Supplementary data are available at *Bioinformatics* online.

## 1 Introduction

In response to stimuli, cells adapt a number of strategies to regulate the signaling pathways of transmembrane receptors, including changing the composition of the plasma membrane (Kusumi *et al.*, 2014), reorganizing the cytoskeleton networks (Liu *et al.*, 2019a) and altering the intermolecular binding kinetics (Kasai and Kusumi, 2014). Receptor tyrosine kinases (RTKs), an important class of transmembrane receptors that control many critical cellular functions such as proliferation, survival and movement (Blume-Jensen and Hunter, 2001; Ullrich and Schlessinger, 1990), are found deregulated in most human cancers (Blume-Jensen and Hunter, 2001). In particular, derailed ‘spatial regulation’ of RTKs can be a hallmark of tumorigenesis or even increased tumor invasiveness (Casaletto and McClatchey, 2012; Salaita *et al.*, 2010; Tomas *et al.*, 2014). These findings suggest that the movement trajectories of RTKs could encode the information of membrane composition and organization, as well as the dimerization and intermolecular binding kinetics of RTKs (Manzo and Garcia-Parajo, 2015; Tsunoyama *et al.*, 2018).

We have previously used the single-particle tracking (SPT) techniques to study the movement of epidermal growth factor receptor (EGFR) in the membrane and showed that the aberrant spatial dynamics of EGFR are associated with the upregulated EphB2 and Src pathways in advanced prostate cancer ([Bibr btab581-B38][Bibr btab581-B39]). In another work, by examining EGFR trajectories in seven breast epithelial cell lines, we developed a physical phenotyping assay termed Transmembrane Receptor Dynamics (TReD) that can assess the metastatic potential of breast cancer cells (Liu *et al.*, 2019a). However, our previous trajectory analysis method was primarily based on extracting the EGFR diffusivity (*D*) and the linear size of the compartment (*L*) from the mean-squared displacement (MSD) plots. While MSD analysis is widely used in the field, much information is lost when turning the raw trajectories into an MSD plot (Kusumi *et al.*, 2005). Although examining individual trajectories and developing novel anomalous diffusion models may provide new insights into the molecular events occurring in the plasma membrane (Dietrich *et al.*, 2002; Fujiwara *et al.*, 2016; Ghosh *et al.*, 2019; Jin *et al.*, 2007; Liu *et al.*, 2016; Zhao *et al.*, 2019), we believe we can also extract hidden features in the membrane from a vast amount of receptor trajectories using deep learning algorithms. Unlike the previous reports that focused on diffusive state characterization using the trajectory-trained machine learning or deep learning models (Dosset *et al.*, 2016; Granik *et al.*, 2019; Matsuda *et al.*, 2018; Wagner *et al.*, 2017), here we directly differentiated cell types based on hidden features extracted from the transmembrane receptor trajectories.

**Fig. 1.**
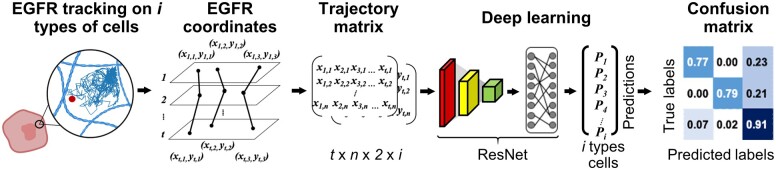
The schematic diagram of deep learning classification of breast cancer cells based on TReD. The deep learning model is a 14-layer variant of a residual neural network (ResNet) optimized using SGD with categorical cross-entropy as the loss function. Our model takes the two-dimensional trajectories of EGFR as inputs and outputs the probabilities of predicted classes, in which the highest probabilities represent the model predictions. The probabilities were then converted into a confusion matrix for a quick assessment of the model prediction

Deep learning approaches, such as convolutional neural network (CNN), and machine learning approaches, such as k-nearest neighbor, have become an increasingly important tool in image-based tumor segmentation and analysis (Campanella *et al.*, 2019; Gautam *et al.*, 2019; Hosny *et al.*, 2018; Ismael *et al.*, 2020; Moen *et al.*, 2019; Trebeschi *et al.*, 2017; Zhao *et al.*, 2018). Assessment and classification of tumors based on tissue microarray images (Shamai *et al.*, 2019), histological images (Araújo *et al.*, 2017), cytological images (Żejmo *et al.*, 2017), and magnetic resonance images (Leithner *et al.*, 2019) have been recently demonstrated. However, none of these characterization methods employed any dynamic information of the live tumor cells or tissues. We believe TReD is not only a new type of physical biomarkers (Liu *et al.*, 2019a,b), but also can be used as input data to train deep learning models, expanding the current arsenal and bringing the cell-based classification of cancer to another level (Fig. 1). Here we demonstrate that we can train a neural network using the motions of EGFR from six breast cancer cell lines and reach 83% accuracy in classifying these cells. Our method is simple, reliable, versatile and cost-effective, and can be combined with the traditional genetic and transcriptomic biomarkers to assess patient-derived tumor samples in the future.

## 2 Results

We first acquired EGFR motions from six breast cancer cell lines which have different gene expression profiles, hormone receptor status and surface receptor status: MCF7, BT474, SKBR3, MDA-MB-468, MDA-MB-231 and BT549. The methods for EGFR labeling and trajectory acquisition were described in our previous publications (Liu *et al.*, 2019a,b) and summarized in the Supplementary Information (SI) Method S1. EGFR was chosen as it is a well-characterized transmembrane receptor which is often overexpressed in carcinogenic cells, thus making it attractive for SPT experiments (Normanno *et al.*, 2006). Based on their receptor status, the six breast cancer cell lines were divided into three subtypes: hormone receptor positive (HR+: MCF7), human epidermal growth receptor positive (HER2+: BT474 and SKBR3) and triple negative (TN: MDA-MB-468, MDA-MB-231 and BT549) (Table 1) (Kao *et al.*, 2009; Neve *et al.*, 2006; Subik *et al.*, 2010). Hormone receptors refer to estrogen (ER) and progesterone receptors (PR), which are often the targets for breast cancer therapy (DeGregorio and Wiebe, 1999; Hudis, 2007). TN subtype refers to breast cancer cells without the presence of ER, PR and HER2. With no primary receptor target for therapy, TN breast tumors have a low survival rate, high recurrence, and high metastatic potential (Liedtke *et al.*, 2008).

**Table 1.** Receptor status for each of the cell lines used in training and testing the model

Cell line	Hormone receptor status		
	ER	PR	HER2
MCF7	+	+	−
BT474	+	+	+
SKBR3	−	−	+
MDA-MB-468	−	−	−
MDA-MB-231	−	−	−
BT549	−	−	−

A total of 13,652 two-dimensional EGFR trajectories of varying track durations [up to 1-min long; 50 ms per time step (*dt*)] were acquired from the six cell lines at 37°C on an Olympus IX-71 inverted fluorescence microscope equipped with a Hamamatsu sCMOS camera (Supplementary Method S1). The acquired raw trajectories were first divided into nonoverlapping 300-*dt* segments and filtered based on the criteria detailed in Supplementary Method S2. In short, we removed the segments of stalled receptors that did not contain any meaningful dynamic information of EGFR. The remaining segments were then repositioned around the coordinate origin, randomly shuffled and stitched into 1,998 of 3,000-*dt*-long ‘reassembled trajectories’ within their cell types (Supplementary Method S2 and Fig. S1). We emphasize that these preprocessing steps were necessary as they guaranteed that each trajectory used for the model training is equally informative. Besides, they preserved the information of both movement speed and pattern of EGFR, which, we believe, hold the key in distinguishing subtypes of cancer cells.

After preprocessing, the 1,998 reassembled trajectories were split into five stratified folds using StratifiedKFold from the scikit-learn library (Pedregosa *et al.*, 2011) (Supplementary Fig. S2 and Method S3), resulting in 20% of trajectory data in each fold. With 5-fold cross-validation, each trial dedicated 4-folds to training (80%) and 1-fold to testing (20%). The 4-folds for training (80%) were further divided into a training set (64%) and a validation set (16%) (Supplementary Table S1). This stratification was employed to keep the representation of each sample (i.e., the label) identical within each fold, ensuring that all samples contribute equally in the model training.

Our model starts with a 14-layer variant of a residual neural network (ResNet) optimized using stochastic gradient descent (SGD) with categorical cross-entropy as the loss function (Supplementary Fig. S3, Method S4 and Method S5) (He *et al.*, 2016). Training time and final performance of neural networks are highly dependent on the choices of a number of hyperparameters, including learning rate (how quickly the model is trained), weight decay (how much the data are regularized), dropout rate (how many units are ignored) and batch size (how many samples are trained in one iteration) (Hutter *et al.*, 2014; Van Rijn and Hutter, 2018). By observing the clues during the early training process, we tuned the hyperparameters based on short runs of a few epochs, eliminating the necessity of running complete grid search or random search (Smith, 2018). The optimized values for the learning rate, weight decay, dropout rate and batch size were found to be 5 × 10^−4^, 5 × 10^−4^, 0.4 and 32, respectively. The model took the 3,000-*dt*-long (150 s) reassembled trajectories as inputs and output a vector containing the probability distributions of predicted classes. The prediction results were visualized in three different ways: a confusion matrix (Supplementary Method S7), a receiver operating characteristic (ROC) curve (Supplementary Method S8) and a uniform manifold approximation and projection [UMAP (McInnes *et al.*, 2018); Supplementary Method S9].

The model was first evaluated using a testing dataset within the StratifiedKFold from the scikit-learn library. The testing dataset was hidden from the network until each fold had completed training. The StratifiedKFold approach ensured each reassembled trajectory to be used in the testing dataset once and used in the training dataset *k* − 1 times. Eventually, five networks (*k* = 5) were trained, and a number of evaluation metrics were obtained based on the testing dataset, providing an assessment of the model performance. Each network generated a set of prediction results, and the model metrics were averaged over the *k* steps.

**Fig. 2.**
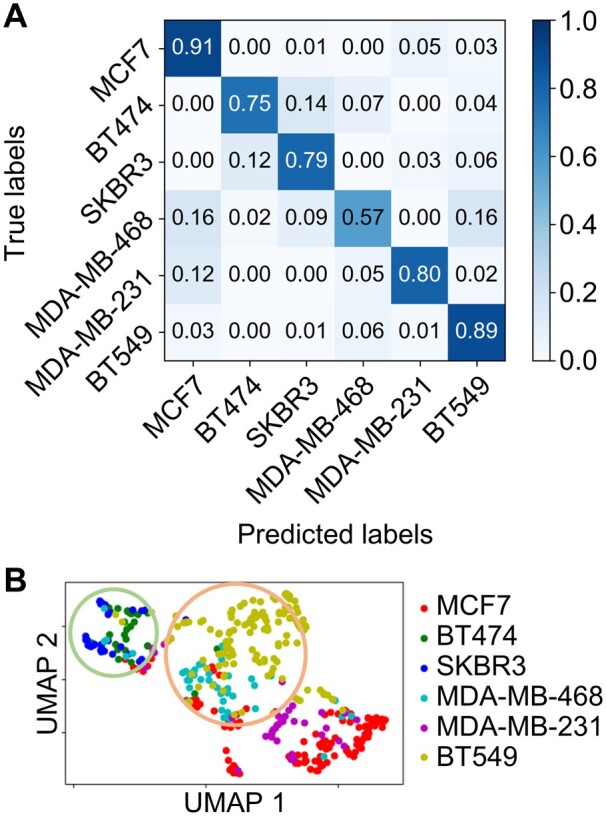
Cell line classification results with an overall accuracy of 83%. (**A**) Normalized confusion matrix showing rates of correct classifications and misclassifications for each cell-line sample (i.e., label). (**B**) UMAP showing the clustering of the low-dimensional projections according to their cell-line origins. The green circle indicates overlapping clusters of the two HER2+ cell lines (BT474 and SKBR3). The orange circle indicates overlapping clusters of the two TN cell lines (MDA-MB-468 and BT549).

Based on the testing set of 396 trajectories from the six cell lines (Supplementary Table S1), our model produced a normalized confusion matrix showing the prediction rates across all the labels and the true positive rates along the diagonal line (Fig. 2A). While the true positive predictions ranging from 0.57 for MDA-MB-468 to 0.91 for MCF7, the overall prediction accuracy was calculated to be 83% for our model (Supplementary Method S7). The worst prediction came from MDA-MB-468 trajectories that were misclassified as MCF7 and BT549 trajectories. The sensitivity and specificity of our model were estimated through the ROC (Supplementary Method S8) (Fawcett, 2006; Grzybowski and Younger, 1997), leading to at least 0.96 AUC (area under the curve) for all labels (Supplementary Fig. S4). Based on their features in the high-dimensional space, the reassembled EGFR trajectories were projected onto a 2D space using the UMAP algorithm (Fig. 2B). While most of the trajectory projections formed distinguishable clusters according to their cell-line origins, notable overlaps between the BT474/SKBR3 clusters (green circle) and between the MDA-MB-468/BT549 clusters (orange circle) were found in Figure 2B.

**Fig. 3.**
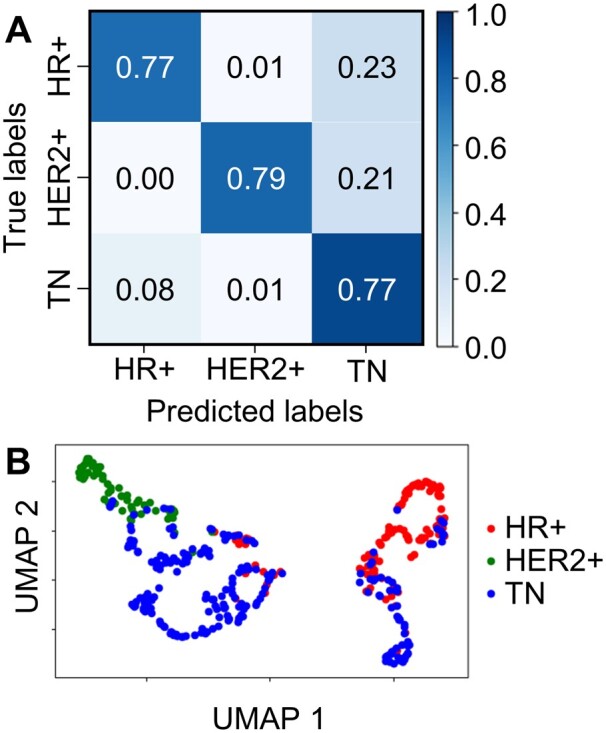
Receptor status classification results with an overall accuracy of 85%. (**A**) Normalized confusion matrix showing rates of correct classifications and misclassifications for each receptor status subtype. (**B**) UMAP showing the clustering of the low-dimensional projections according to their receptor status.

The model was also trained and tested based on receptor status of the six cell lines (HR+: MCF7, HER2+: BT474 and SKBR3 and TN: MDA-MB-468, MDA-MB-231 and BT549 in Table 1). The resulting confusion matrix had the true positive rates ranging from 0.77 to 0.90 for the three labels, with an overall accuracy of 85% (Fig. 3A, Supplementary Method S7). The AUC of ROC curves was at least 0.94 for all labels (Supplementary Fig. S5). UMAP was again used to visualize the clustering of low-dimensional projections that preserve the local and global structures in the high-dimensional feature space. Interestingly, the projections from the TN cell trajectories formed two clusters that overlapped with the HER2+ and HR+ clusters, respectively (Fig. 3B). The result of overlapping clusters reflected the high misclassification rates seen in the confusion matrix: 0.23 for HR+ cells and 0.21 for HER2+ cells were misclassified as TN cells.

**Fig. 4.**
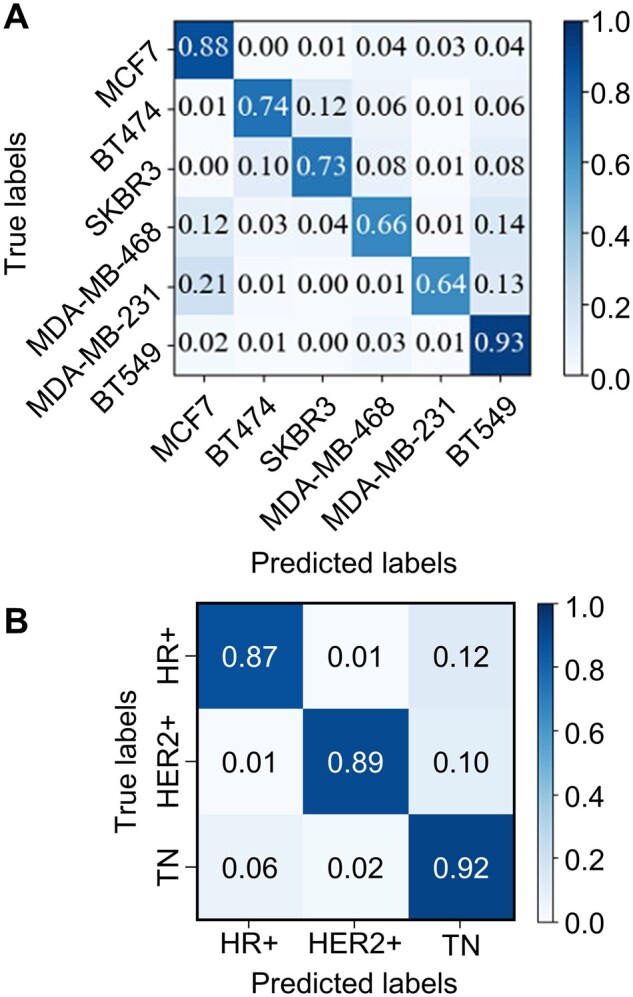
Validation of the trained models using a newly reassembled dataset. The classification of six cell lines and receptor statuses achieved 82% and 90% overall accuracies, respectively. (**A**) Confusion matrix on the new dataset, using the pretrained model from Figure 2. (**B**) Confusion matrix on the new dataset, using the pretrained model from Figure 3.

To answer the question of whether or not the segment-stitching step in the data preprocessing generated any bias or artifact during the model training, we randomly shuffled the segments again and created a new set of 1,998 reassembled trajectories. When testing the trained models on this new dataset (1,998 trajectories), we obtained two confusion matrices (Fig. 4) that were similar to the matrices obtained from the previous testing dataset (396 trajectories, Figs 2A and 3A). The overall prediction accuracies were 82% for the six cell lines and 90% for the three receptor status groups. Interestingly, the misclassification rates from MDA-MB-231 to MCF7 and from MDA-MB-231 to BT549 noticeably increased on the new dataset (from 0.12 to 0.21 and from 0.02 to 0.13, respectively) while the true positive rate of MDA-MB-231 prediction dropped from 0.81 to 0.64 (Figs 2A and 4A). In contrast, while high misclassification rates were still seen in the confusion matrix of receptor status prediction, those rates were nearly halved on the new dataset: 0.12 for HR+ cells and 0.10 for HER2+ cells were misclassified as TN cells. As a result, the true positive rates became higher (>0.87) on the new dataset (Fig. 4B). While both HR+ and HER2+ cells had ∼0.10 rate to be misclassified as TN cells, the misclassification rates between HR+ and HER2+ cells were ∼0.01, similar to the previous testing results (Fig. 3A). These similarities in the confusion matrices indicated that our trained models are reliable in classifying cell types and receptor status, regardless of the shuffling order.

**Fig. 5.**
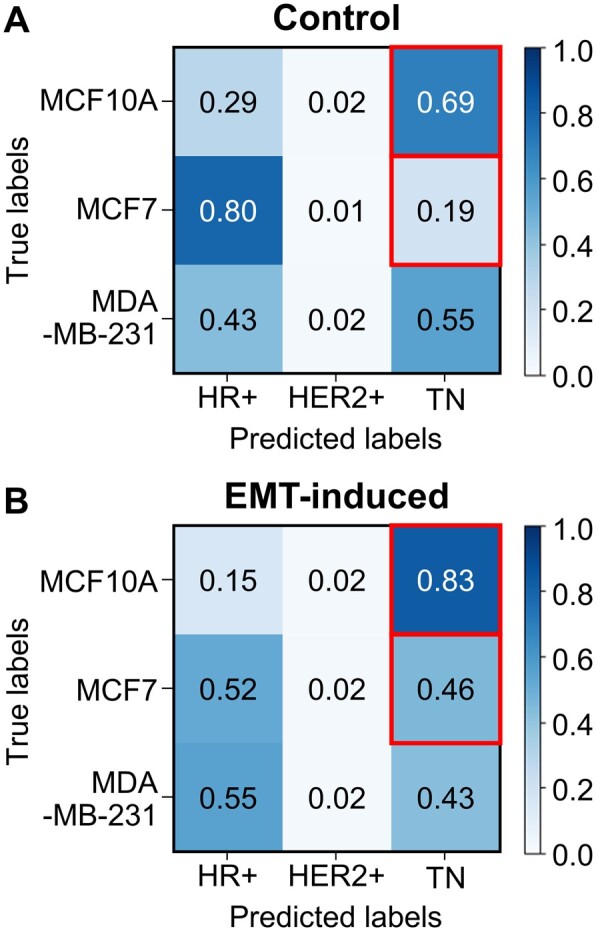
Receptor status classification using the trained model before and after the induction of EMT. MCF10A and MCF7 increased in TN classification by 20% and 142%, respectively. MDA-MB-231 decreased in TN classification by 22%. (**A**) Confusion matrix of the three cell lines before the EMT induction. (**B**) Confusion matrix of the three cell lines after the EMT induction. After EMT induction, both MCF10A and MCF7 cells have even higher chances to be classified as TN cells (red squares).

To further investigate the capabilities of the trained models, we tested EGFR trajectories from MCF10A cells (benign breast epithelial cells) with and without the induction of epithelial–mesenchymal transition (EMT, Supplementary Method S1). MCF10A cells are nonmalignant breast epithelial cells that are commonly used in comparison with MDA-MB-231 and other breast cancer types in physical oncology research (Kumar *et al.*, 2018). Using MCF10A as a control, we wanted to see if our model (which was trained only on cancerous cells) can truly classify phenotypic changes such as EMT. EMT induction could transform benign cells and noninvasive cancer cells into highly aggressive cells (Scheel *et al.*, 2011), thus resulting in an increase in TN classification of EMT-induced MCF10A. Together with the trajectories from MCF7 cells (HR+) and MDA-MB-231 cells (TN), as well as their EMT-induced cells, we performed the receptor status classification using the trained model (Fig. 5). We emphasize that these trajectories were independently acquired and never used in the training process. As expected, all three cell lines had low chances (<0.05) to be misclassified as HER2+ cells. In contrast, MCF10A (benign) and MDA-MB-231 (TN) cells had high chances to be misidentified as TN and HR+ cells, respectively, before EMT induction (Fig. 5A). After EMT induction, both MCF10A (benign) and MCF7 (HR+) cells had much higher rates to be classified as TN cells (20% and 142% increase from their original values, respectively), while fewer MDA-MB-231 cells were classified as TN cells (22% decrease, Fig. 5). These results indicated that, after EMT induction, both MCF10A (nontumorigenic) and MCF7 (noninvasive) cells produced EGFR trajectories that resembled those on the TN cells (highly metastatic), resonating with the previous result that the diffusivity of EGFR increased in MCF10A and MCF7 cells after EMT induction (Liu *et al.*, 2019a). While the signature of EMT could not be fully captured by the trajectory features that normally differentiate among the HR+, HER2+ and TN subtypes, our results indicated that TReD discerns the changes caused by epithelial cell transition.

## 3 Discussion

We demonstrated that well-trained deep learning models based on ResNet (Supplementary Fig. S3 and Method S5) can differentiate EGFR trajectories from six breast cancer cell lines (Fig. 2) and from three groups of cells with distinct receptor status (HR+, HER2+ and TN, Fig. 3) with high accuracies (83% and 85%, respectively). In this report, we use the terms ‘trajectory classification’ and ‘cell classification’ interchangeably, as the probabilities to identify the cell origin of a trajectory should be identical to the probabilities to identify the cell itself (assuming each cell produces tens to hundreds of EGFR trajectories for analysis). Our method is reliable, as the control experiment also showed equally good overall classification accuracies (82% and 90%, respectively, Fig. 4). To the best of our knowledge, this is the first time that the EGFR trajectories are used as inputs to train deep learning models that differentiate breast cancer cells with distinct metastatic potential. While other machine learning classifications of single-particle trajectories focused on the identification of diffusive modes [e.g., Brownian diffusion, confined diffusion or directed diffusion (Dosset *et al.*, 2016; Matsuda *et al.*, 2018; Wagner *et al.*, 2017)] or diffusive conditions [e.g., continuous time random walk and fractional Brownian motion (Granik *et al.*, 2019; Muñoz-Gil *et al.*, 2019)], and used simulated trajectories for training, we aimed to directly differentiate the cell types and only used the experimental EGFR trajectories for our model training.

The members of EGFR family are able to form homodimers or heterodimers (Alroy and Yarden, 1997), and the density of the receptors does affect the probability of dimerization and the diffusivity of receptors (Chung *et al.*, 2010). We analyzed the mRNA expression levels of EGFR family, including EGFR, HER2, HER3 and HER4, using the same set of databases on the *Genevestigator* (Supplementary Fig. S6). The results did not show a strong negative correlation between the EGFR expression level and the measured diffusivity across these six cell lines we tested, and the correlation coefficient of EGFR expression and EGFR diffusivity was −0.09. Regarding HER2 expression level, the result showed the SKBR3 and BT474 cells with the highest HER2 gene expression levels (10-fold higher than other cells) exhibited the lowest EGFR diffusivities. This result cohered with Agazie’s research which demonstrated that HER2 overexpression enhances the stability of HER2 homodimers and HER2-EGFR heterodimers (Hartman *et al.*, 2013). In addition, HER3 may also play a similar role in the formation of HER3-EGFR heterodimers, which reduces the diffusivity of EGFRs.

It is well known that the highly invasive breast cancer cell lines, such as MDA-MB-231 and BT-549, show traits of EMT with upregulated EMT markers (Blick *et al.*, 2008; Choi *et al.*, 2013). EMT involves a series of signaling pathways that alter the morphology of the cell to become more migratory and metastatic in nature. The process involves multiple biochemical changes, including the expression of specific cell-surface proteins, the reorganization of cytoskeletal networks, and the degradation of extracellular matrix (Kalluri and Weinberg, 2009). As we believe the information about plasma membrane organization, composition, receptor interactions, and receptor status are embedded in the TReD, it is thus important to test whether or not our neural network model can capture the signature of EMT induced in the benign and the noninvasive cancer cells. As we expected, after EMT induction, both MCF10A (benign) and MCF7 (noninvasive) cells behaved more like TN cells (Fig. 5). Interestingly, the TN cell line, MDA-MB-231, became less TN-like after EMT induction. This result indicated that the current model cannot fully capture the EMT signature. One possibility is that many EMT-related trajectory features were not included in the features that normally differentiate among the three receptor status groups. Another possibility is the EMT induction produced very heterogeneous responses among single cells (i.e., not all cells being successfully transformed into aggressive phenotypes). Our next goal is to use other methods to induce invasiveness, such as the use of macrophage coculture system to activate the JNK and NF-κB pathways (Hagemann *et al.*, 2005), and test our models again. Our current model training excluded the trajectories from MCF10A cells, thus resulting in a high misclassification rate of MCF10A as TN cells, even before the EMT induction (Fig. 5A). We have attempted to train a model with MCF10A trajectories, but MCF10A was always misclassified as MDA-MB-231 at a high rate. The reason is not clear at this moment. Other than the breast cancer cell lines, we are extending our method to classify prostate cancer cell lines and will publish that result in the future.

Our method is not only reliable and versatile, but also simple and cost-effective as the data can be preprocessed, and the networks can be trained on a PC with GPU (Supplementary Method S4). It takes less than an hour to preprocess 13,652 EGFR raw trajectories, approximately 2 hours to train, validate and test the model, and less than 5 min to test the new set of 1,998 assembled trajectories. With a high classification accuracy demonstrated on the cell lines, it is very promising that this biophysical assessment platform can rapidly and precisely predict the metastatic potential of patient-derived samples, such as circulating tumor cells (CTC) or tumor organoids. For instance, EGFR tracking can be integrated with a microfluidic CTC capture system, so the combination of CTC capture and the SPT systems could provide a unique chance to achieve streamlined physical and molecular analyses on the same CTCs. We also anticipate the integration of our deep learning models with single-cell transcriptomic analysis of CTCs in the near future.

As our trajectory-based deep learning classification is inherently an image-based single-cell differentiation method (i.e., trajectories can be collected from individual cells in a tumor sample), it can be extended to investigate single-cell behaviors in the tumor microenvironment and tumor heterogeneity (Gupta *et al.*, 2011; Provenzano *et al.*, 2009). As cell culture, labeling and imaging can all be carried out by automation and 10^1^–10^3^ of independent receptor trajectories can be obtained in one field of view, more comprehensive model training and better classification accuracy are expected in the near future. Whereas machine learning classifications of cancer cells are primarily focused on the label-free images of cell morphology (Chen *et al.*, 2016) and the fluorescence images of cell organelles or cytoskeletal structures (Jones *et al.*, 2009), here we demonstrate an alternative way to classify cancer cells using a dynamic, biophysical feature that is easy to acquire.

## Funding

This work was supported by the Cancer Prevention & Research Institute of Texas [RR160005 to T.E.Y.]. T.E.Y. is a CPRIT Scholar in Cancer Research. H.-C.Y. acknowledges the support of this work by Texas 4000, the Robert A. Welch Foundation [F-1833], National Institutes of Health [GM129617] and National Science Foundation [2029266]. Y.-L.L. is a recipient of the YingTsai Young Scholar Award of China Medical University [CMU108-YTY-01] and also a recipient of the Young Scholar Fellowship Program from the Ministry of Science and Technology in Taiwan [MOST 108-2636-E-039-001].

*Conflict of Interest*: none declared.

## Data availability

The dataset and a python implementation of deep learning-based classification can be found at https://github.com/soonwoohong/Deep-learning-for-EGFR-trajectory-classification.

## Supplementary Material

btab581_Supplementary_Data
