# Supplementary material for: Deep learning-based classification of breast cancer cells using transmembrane receptor dynamics
Source: Bioinformatics. 2021 Aug 15;38(1):243–9. doi: 10.1093/bioinformatics/btab581 (PMC8696113; doi:10.1093/bioinformatics/btab581)
Supplement: btab581_Supplementary_Data [file btab581_supplementary_data.docx]

**Supplementary Information**

**Deep Learning-Based Classification of Breast Cancer Cells Using Transmembrane Receptor Dynamics**

Mirae Kim^1,†^, Soonwoo Hong^2,†^, Thomas E. Yankeelov^2,3,4,5,6^, Hsin-Chih Yeh^2,7,^* and Yen-Liang Liu^8,^*

^1^Department of Computer Science, Rice University, Houston, TX 77005, USA, ^2^Department of Biomedical Engineering, ^3^Oden Institute for Computational Engineering and Sciences, ^4^Department of Diagnostic Medicine, ^5^Department of Oncology, ^6^Livestrong Cancer Institutes, ^7^Texas Materials Institute, University of Texas at Austin, TX 78712, USA, ^8^Master Program for Biomedical Engineering, and ^9^Graduate Institute of Biomedical Sciences, China Medical University, Taichung 40678, Taiwan.

*To whom correspondence should be addressed.

^†^These authors contributed equally to this work.

**Table of Contents**

METHOD S1 | Single-particle tracking and experimental conditions 3

METHOD S2 | Preprocessing of the raw trajectories 4

METHOD S3 | Data labeling and division 5

METHOD S4 | Hardware and software 6

METHOD S5 | Model architecture and training 6

METHOD S6 | Model cross validation and testing 7

METHOD S7 | Confusion matrix and overall accuracy 7

METHOD S8 | ROC and AUC 8

METHOD S9 | UMAP 9

METHOD S10 | Comparisons with other deep learning networks. 9

TABLE S1 | Data division by cell line, receptor status, and dataset after preprocessing 11

FIGURE S1 | Schematic of the preprocessing method 12

FIGURE S2 | Example trajectories after preprocessing 13

FIGURE S3 | Model Architecture 14

FIGURE S4 | ROC curves of cell-line classification 15

FIGURE S5 | ROC curves of receptor status classification 16

FIGURE S6 | Correlations between EGFR diffusivity and expression levels of EGFR family 18

FIGURE S7 | Cell line classification results with and without dropout layer 19

TABLE S2 | Comparison results with different network architectures 20

FIGURE S8 | Model architectures for comparison results 21

FIGURE S9 | Confusion matrix with different model architectures. 22

Supporting References 23

# METHOD S1 | Single-particle tracking and experimental conditions

**Cell culture conditions.** The SPT TReD data were gathered using previously described methods (Liu, et al., 2019). For BT474, SKBR3, MDA-MB-231, MDA-MB-468, and BT549, the cells were incubated in DMEM/F12 with 10% fetal bovine serum (16140071, Thermo Fisher Scientific) and 50 U/mL penicillin-streptomycin (15070063, Thermo Fisher Scientific). For MCF7, the cells were incubated in MEM (11095-080, Thermo Fisher Scientific) supplemented with 10% fetal bovine serum and 50 U/mL penicillin-streptomycin. For MCF10A, the cells were incubated in DMEM/F12 medium supplemented with 5% horse serum (16050122, Thermo Fisher Scientific), 20 ng/mL epidermal growth factor (AF-100-15, Peprotech), 0.5 μg/mL hydrocortisone (H0888, Sigma-Aldrich), 100 ng/mL cholera toxin (C8052, Sigma-Aldrich), 10 μg/mL insulin (I9278, Sigma-Aldrich), and 50 U/mL penicillin-streptomycin.

**Cell labeling.** The cells were grown in an optical imaging 8-well chambered coverglass (155409, Thermo Fisher Scientific) until 70% confluency, then stained using 1:1000 dilution of Hoechst 33258 (H3569, Thermo Fisher Scientific) for 10 minutes at 37ºC. Then, the cells were labeled with 100pM Anti-EGFR IgG antibody-conjugated fluorescent nanoparticles, diluted from a 30nM solution of Biotinylated monoclonal anti-EGFR antibodies (Clone Ab-3, MS-311-B, Thermo Fisher Scientific) and 40 nm NeutrAvidin-labeled red fluorescent nanoparticles (F8770, Thermo Fisher Scientific) in 1.5% bovine serum albumin (BSA, S7806, Sigma-Aldrich) mixed 1:1 in PBS, for 10 minutes at 37 ºC. After 10 minutes, the labeling solution was removed then the wells were washed twice and filled with 200 µl of PBS. The cells were then put on the microscope immediately with a temperature-controlled stage (Stable Z System, Bioptechs) at 37 °C.

**Epithelial-mesenchymal transition (EMT) induction.** Three breast cancer cell lines, MCF10A, MCF7, and MDA-MB-231, were induced with epithelial-mesenchymal transition (EMT) to simulate dysregulated membrane environment (Liu, et al., 2019; Thiery, et al., 2009), and its effect on EGFR trajectories and classifications. EMT induction was done using EMT induction medium (StemXVivo EMT Inducing Media Supplement). The cells were grown for two days in the EMT induction medium and then starved for one day before the EGFR tracking experiments.

**Single-particle tracking.** The tracking was done with an Olympus IX-71 inverted fluorescence microscope equipped with a 60x 1.2 N.A. water objective (UPLSAPO 60XW, Olympus). A metal halide lamp with a 545/25 nm bandpass excitation filter was used to provide wide-field excitation, and the emission was collected by a Scientific CMOS camera (ORCA-Flash4.0, Hamamatsu) with 565 nm dichroic and 605/70 bandpass filter.

The series of images were transformed into a series of binary pixel images by identifying those pixels that have intensities greater than three standard deviations compared to the background using DIPimage MATLAB toolbox or those that are above a certain threshold. Then a high pass filter and a 2D Gaussian filter with σ of 5 pixels. Within the 5 pixels, the highest intensity pixel was chosen as the starting point, which then was fitted using the Gaussian fitting method in a surrounding 2σ_xy_ x 2σ_xy_ square, with σ_xy_ as the 2D Gaussian approximation of the point spread function. The starting point was updated until convergence with less than 10^-5^ pixel movement. The coordinates were cross-correlated to verify the Gaussian fitting and were only recorded as coordinates if achieving a cross-correlation value of 0.7 or more.

Images were gathered at 20 frames per second for one minute, totaling 1,200 frames. With diffusivity *D*, the probability of the particle traveling a distance greater than *d* in time *Δt* is:

| $P\left( d,\Delta t \right)=exp\left[ \frac{-d^{2}}{4D\Delta t} \right]$ | (1) |
| --- | --- |

With 3D coordinates of (x,y,t) from the images, coordinates at *t*  and *t+Δt* were used to calculate the probability using **Eq. 1** (Saxton, 1993). If the resulting probability is greater than 0.05, then the two points are connected as a trajectory. This process then results in several short trajectories. With the series of short trajectories, the end coordinates of one short trajectory and the starting coordinates of all later short trajectories are compared using Eq.1 to determine if the probability is greater than 0.01. With multiple later trajectories with a probability greater than 0.01, the trajectory with the smallest *Δt* is connected. This process repeats until all the short trajectories that satisfy the probability condition have been connected, which then the connected trajectories are exported as a series of coordinates with a maximum length of 1200. We received this method as a gift from Prof. Keith Lidke at the University of New Mexico.

# METHOD S2 | Preprocessing of the raw trajectories

The data went through preprocessing to ensure that the model is trained on representative and informative data. The experimental TReD trajectories were gathered for up to a minute each, with 50 ms timestep (dt), resulting in a maximum of 1200 pairs of x and y coordinates in each trajectory. Then each trajectory was cut into four sub-trajectories with 300 timesteps each, and the sub-trajectories were randomized within each label. The sub-trajectories then were filtered for incomplete segments and immobile segments. The incomplete segments were filtered by detecting for consecutive zeros in the coordinates since trajectories with fewer than the maximum number of coordinates are padded with zeros. The immobile segments were filtered by calculating the mean square displacement (MSD) of the segments at every 5-*dt* using (Liu, et al., 2019):

| $MSD\left( \Delta t \right)=\left\langle{(\boldsymbol{r}\left( t+\Delta t \right)-\boldsymbol{r}(t))}^{2} \right\rangle$ | (2) |
| --- | --- |

All the MSDs for each segment were averaged to be compared to the threshold of 0.001 μm^2^. Then, each segment’s minimum in *x* and *y* were subtracted from each of the coordinates to relocate the trajectories near the origin, with each trajectory’s minimum *x* and *y* value being zero. The relocation preserved the dynamic data included in the velocity and the shape of the trajectories while minimizing bias caused by the location of the trajectories within the image. The segments were then randomly assembled into groups of 10 within the same label, resulting in assembled trajectories with a length of 3000-*dt*. Since the individual receptor dynamics measured within the time frame and the image frame can vary from one receptor to another even from the same cell while the overall dynamics showing a pattern between the cell types, the shuffled reassembly of sub-trajectories within the same label distributed the features found across the trajectories. This would ensure that each 3000-dt input to the model would have information regarding the overall features of the cell type measured, not just information from a single receptor or a single cell.

# METHOD S3 | Data labeling and division

The breast cancer cell lines used to train, validate, and test the model were MCF7, BT474, SKBR3, MDA-MB-468, MDA-MB-231, and BT549. The cell lines were divided into subtypes based on the known expression of hormone and membrane receptors (**Table S1**). Those with both estrogen and progesterone receptors present were considered as hormone receptor-positive (HR+; MCF7). Those with upregulated human epithelial growth factor 2 (HER2) receptor present were considered as HER2-positive (HER2+; BT474 and SKBR3). Those with no significant level of estrogen, progesterone, or HER2 receptor present were considered as triple-negative (TN; MDA-MB-468, MDA-MB-231, and BT549) (Kao, et al., 2009; Neve, et al., 2006; Subik, et al., 2010).

# METHOD S4 | Hardware and software

All preprocessing and model training/testing were done using custom-built PC with Intel® Core™ i7-9700K CPU @ 3.60GHz and NVIDIA RTX 2080Ti. The NVIDIA graphics processing unit (GPU) provided 4352 CUDA Cores with a standard memory of 11GB GDDR6 and memory bandwidth of 616 Gb/s. The data were preprocessed using MATLAB R2019a and were formatted as comma-separated values (.csv) files. The model was built, trained, and tested using Python (version 3.7.3). NumPy (version 1.16.4) was used for data loading and reshaping. Keras (version 2.2.4) and TensorFlow (version 1.14.0) were used to build, train, and test the models. Accuracy and loss per epoch plots were generated using matplotlib (version 3.1.0). Result visualization using confusion matrix was performed using Scikit-learn (version 0.21.2), and k-fold stratified cross validation was employed using StartifiedKFold from the scikit-learn library. UMAPs were created using umap-learn (version 0.3.10).

# METHOD S5 | Model architecture and training

The TReD based deep learning model is a derivative of deep CNN-based residual neural network, ResNet (He, et al., 2016), implemented to classify epithelial growth factor receptor (EGFR) trajectories on breast cancer cells into specific cell lines or subtypes. Compared to other deep CNN-based network, One of the advantages from ResNet is that one can build multiple convolution layers without overfitting using so-called residual connection to highly improve the model performance. The model defines a convolution block to consist of three one-dimensional (1D) convolution layers with one 1D convolution layer in the shortcut, with batch normalization and rectified linear unit (ReLU) activation. The model defines an identity block to consist of three 1D convolution layers with the linear shortcut, also with batch normalization and ReLU. The pathways (convolution and shortcut) are added right before the last ReLU of the block. The model initializes with a 1D convolution layer with batch normalization and ReLU, followed by the convolution block, the identity block, and, again, the convolution block. The model then proceeds to the 1D spatial dropout layer to decrease overfitting, followed by last 1D convolution layer, ReLU, 1D global average pooling, and dense and softmax layers, eventually leading to the output of probabilities of predicted classes. As employing batch normalization and dropout layers simultaneously in the model might cause bias in prediction (Li, et al., 2019), we inserted only one dropout layer between the last batch normalization layer and the final softmax layer. By doing so, we not only minimized the overfitting problem but also slightly improved the model accuracy (**Fig. S7**).

The model loss was calculated using the categorical cross-entropy loss function from the keras library:

|  | $h\left( p,t \right)= -\sum t_{i}log(p_{i})$ | (3) |
| --- | --- | --- |

The loss, *h(p,q)* was calculated using the truth (1.0 or 0.0), *t*, and the predicted probability, *p*, within each label, *i*. The loss was minimized using a stochastic gradient descent (SGD) optimizer with momentum of 0.01.

Early stopping was implemented to preserve the best model conditions before overfitting. The metric was validation loss, which was to be minimized (mode = ‘min’) at all times within 50 epochs (patience = 50). With no improvements in validation loss observed within 50 epochs, the model parameters would be set as best, and the training process would stop.

# METHOD S6 | Model cross validation and testing

To avoid overfitting and improve model performance, *k*-fold stratified cross validation was employed using StratifiedKFold from the scikit-learn library. Overfitting is one of the biggest problems in deep learning, which refers to a model that learns every detail on the training sets and has a bias to the training sets. *k*-fold cross validation helps to generalize a model because it resamples training set and evaluate the model on new data. The testing set was determined by stratified *k*-fold to account for the uneven sizes of each classification. The stratified *k*-fold ensures even proportion of data in each training, validation, and test set in each of the folds. We chose *k* = 5, and therefore five folds were used to separate between test and training sets, which then the other five-folds were used to separate testing and validation set within the testing set. The final proportions of data were 64%, 16%, and 20% for training, validation, and testing, respectively. *k* models (*k* = 5) are trained to compute the mean metrices on the testing dataset, which represents the overall performance of our model. Each model generates the prediction results, and the model metrics are then averaged over the *k* steps. The prediction results in the ensemble was determined as the average prediction results on the *k* test sets.

# METHOD S7 | Confusion matrix and overall accuracy

A confusion matrix is a table that visualizes the model performance on test data. It also helps to understand the way the classifier predicts and which class it misclassifies. The normalized confusion matrix depicts the true positive rate in each label. For *n* classifications with true label *i* and predicted label *j*, the normalized confusion matrix was calculated using:

|  | $\frac{N_{j=i}}{\sum_{i=1}^{n} N_{i}}$ | (4) |
| --- | --- | --- |

The element (*i*, *j*) of each confusion matrix shows the probability of predicted label *j* given that the ground truth was label *i*. Thus, the values of diagonal elements represent how accurately a model classifies a label *i*. The higher we can get the diagonal values, the better the model classifies the test data.

The overall accuracy was calculated by taking sum of the true positive rate of each label and multiplying them by the corresponding label’s data proportion in the dataset. By multiplying by the data proportion, it minimizes the bias caused by the inconsistent number of trajectories per label. This was done using the accuracy_score function from scikit-learn library. Since the sample numbers for each class are different (**Table S1**), we should apply the weights on the elements of the confusion matrix. Our testing data (total number = 197) consist of 57 MCF7 (29%), 14 BT474 (7%), 17 SKBR3 (9%), 22 MDA-MB-468 (11%), 20 MDA-MB-231 (10%), and 67 BT549 (34%). On the confusion matrix, the diagonal elements depict 91%, 75%, 79%, 80%, and 89% for MCF7, BT474, SKBR3, MDA-MB-468, MDA-MB-231, and BT549, respectively. Therefore, the overall accuracy can be calculated as 0.29·91 + 0.07·75 + 0.09·79 + 0.11·57 + 0.10·80 + 0.34·89 ≈ 83.

# METHOD S8 | ROC and AUC

Receiver operating characteristic (ROC) curve and area under the curve (AUC) relate sensitivity and 1-specificity for threshold determination. They were employed as another visualization method to show our model performance. The sensitivity and 1-specificity are calculated as true positive rate and false-positive rate, respectively. With each point of the ROC curve displaying the tradeoff between sensitivity and 1-specificity, the linear ROC represents random guessing probability between true and false, 0.50. The AUC of the ROC curve indicates the probability of correctly assigning a higher probability to the true sample if given two samples from different classifications. Because ROC evaluates binary cases (true or false) pertaining to one label, each classification label was represented by a separate curve. As a reference, we added *specificity = 0.8, 0.9*, and *1.0* (horizontal), and *1-sensitivity* = *0.0*, *0.1*, and *0.2* (vertical) lines.

# METHOD S9 | UMAP

Uniform manifold approximation and projection (UMAP) is a novel algorithm for dimension reduction for visualization. We employed UMAP to visualize the clustering in the low-dimensional projections that preserve local and global structure in the high-dimensional feature space. UMAP is a nonlinear dimension reduction technique that allows researchers to visualize high-dimensional data in 2D or 3D spaces (McInnes, et al., 2018). The last hidden layer representation was collected for applying UMAP. Each dot colored by a corresponding label represents one trajectory as embedded by UMAP. We used umap-learn library with the default hyperparameters: n_neighbors = 15, min_dist = 0.1, n_components = 2, metric = ‘euclidean’.

# METHOD S10 | Comparisons with other deep learning networks.

In **Table S2**, we compared different deep learning network architectures. As we described in **Method S5** and **Method S6**, early stopping and 5-fold cross-validation were employed for this comparison. Thus, each training would automatically stop, and the model parameter would be set as best when no improvements over 50 epochs are observed. The results shown in **Table S2** summarized the mean estimates of each architecture from cross-validation.

We first tested the different numbers of ResNet blocks. Three ResNet-variant networks have different numbers of convolutional blocks. Our base model has two convolutional blocks (C) with one identity block (I), termed “CIC.” Another ResNet has more simple structure with only one convolutional block (C), and the other has more layers with CICIC structures (with three convolutional blocks). They shared similar model accuracy and training time, but ours showed the best performance among other ResNet variants. We would like to highlight that there is a trade-off between the model complexity and overfitting. It means that too many layers could improve the accuracy, but it might also cause overfitting. There was not much difference in accuracy among ResNet variant models, but we could also observe this phenomenon. In other words, CIC architecture (ours) outperforms the simple ResNet with a single convolutional block, but more layers (CICIC) decreased the accuracy because of the over-fitted model. With the CICIC model, the false classification as MCF7 or BT549 was slightly higher than that of the CIC model.

However, too much simple models, such as AlexNet (Krizhevsky, et al., 2012), and VGGNet (Simonyan and Zisserman, 2014), could not achieve the acceptable level of accuracy (<70%). As seen in **Figure S9**, the predictions made by these two models were poor. This is mainly because the complexity of these models was too low and led to underfitting. Batch normalization in the AlexNet-variant network made a small improvement over VGGNet-variant one, but it was not sufficient to outperform ResNet-variant models.

By doing comparison tests, we could also see the LSTM (Long short-term memory)-based model was the worst model in terms of both accuracy and training time. This was quite interesting because LSTM is broadly known as a good way for training and predicting 1D datasets (Gers and Schmidhuber, 2000; Gers, et al., 2002). We pointed out that this was attributed to the fact that our datasets are different from normal 1D data; our datasets have a long 3,000 time-step. This not only makes it difficult to extract meaningful features from the data but also increases the training time. The high dimensionality and complexity of data made the difference compared to the CNN-based models, including ResNet, AlexNet, and VGGNet (Yin, et al., 2017).

**
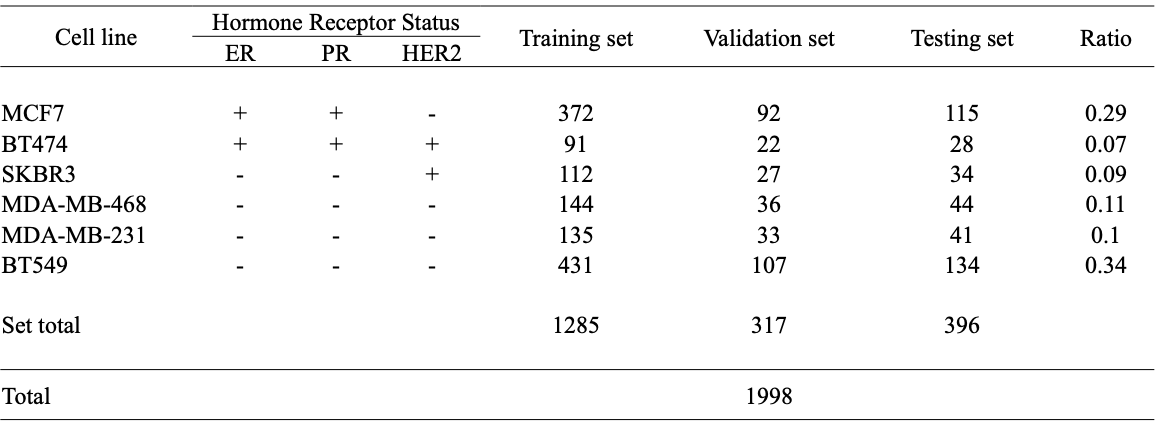
**

# TABLE S1 | Data division by cell line, receptor status, and dataset after preprocessing

After preprocessing, the dataset consisted of 29% MCF7, 7% BT474, 9% SKBR3, 11% MDA-MB-468, 10% MDA-MD-231, and 34% BT549. Due to the varying number of trajectories in each label, the division between training, validation, and testing were done using stratification, preserving the rate of each label within each dataset. During receptor status classification and testing, the cell lines were labeled according to their receptor status: HR+; MCF7, HER2+; BT474 and SKBR3, and TN; MDA-MB-468, MDA-MB-231, and BT549.

**
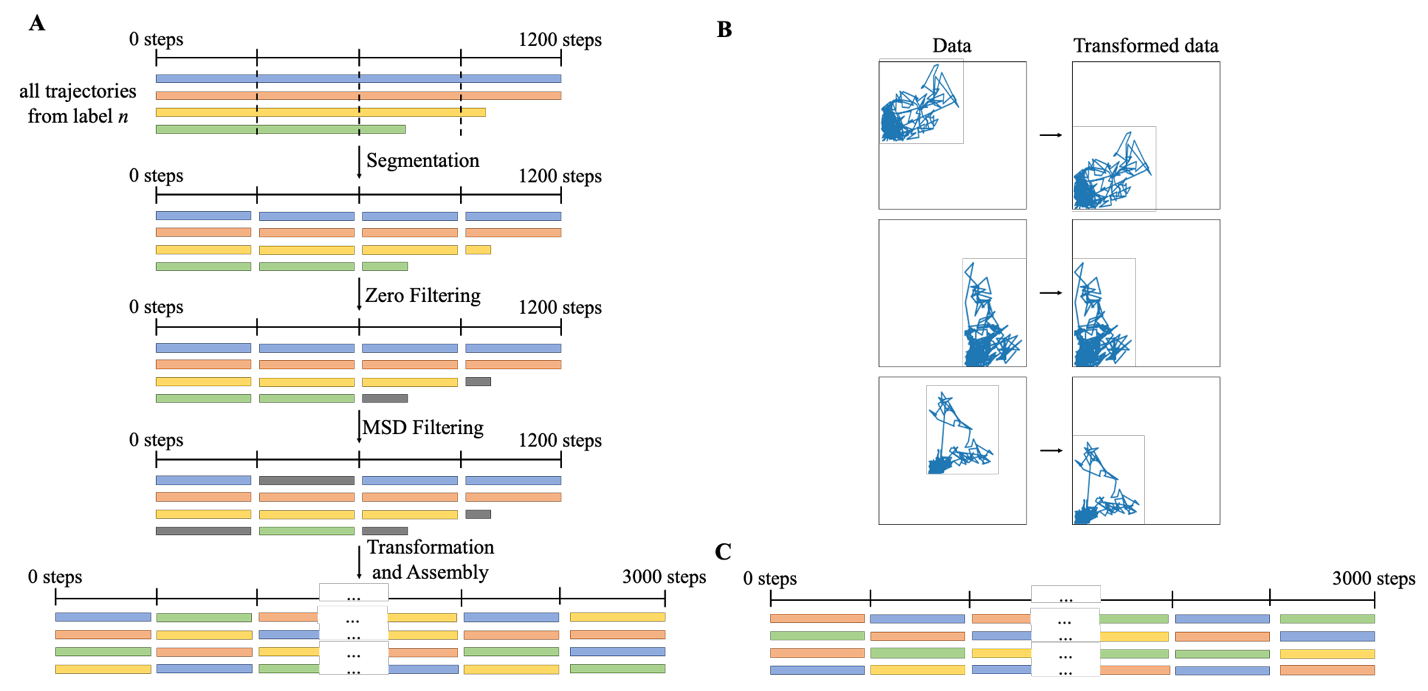
**

# FIGURE S1 | Schematic of the preprocessing method

**(A)** Overall diagram of the preprocessing method. First, the 1200-*dt* trajectories are cut into four segments of 300-*dt* through segmentation. Then, the segments are filtered if meeting either condition: (1) padded with zeros at the end of the segment, indicating that the segment did not have the maximum number of coordinates, (2) the segments contain evidence of immobility. Immobility was defined as having MSD less than 0.001 μm^2^ (see **METHOD S2**). Lastly, the segments were transformed to relocate to the origin by subtracting minimum x and y coordinates from each of the coordinates. The preprocessed segments were then randomly assembled in groups of 10 to form assembled 3000-*dt* trajectories. **(B)** Transformation of segments to relocate near the origin. **(C)** Visualization of the re-shuffled and re-assembled testing set with randomized segments compared to the original training and testing set in **Fig. S1A.**


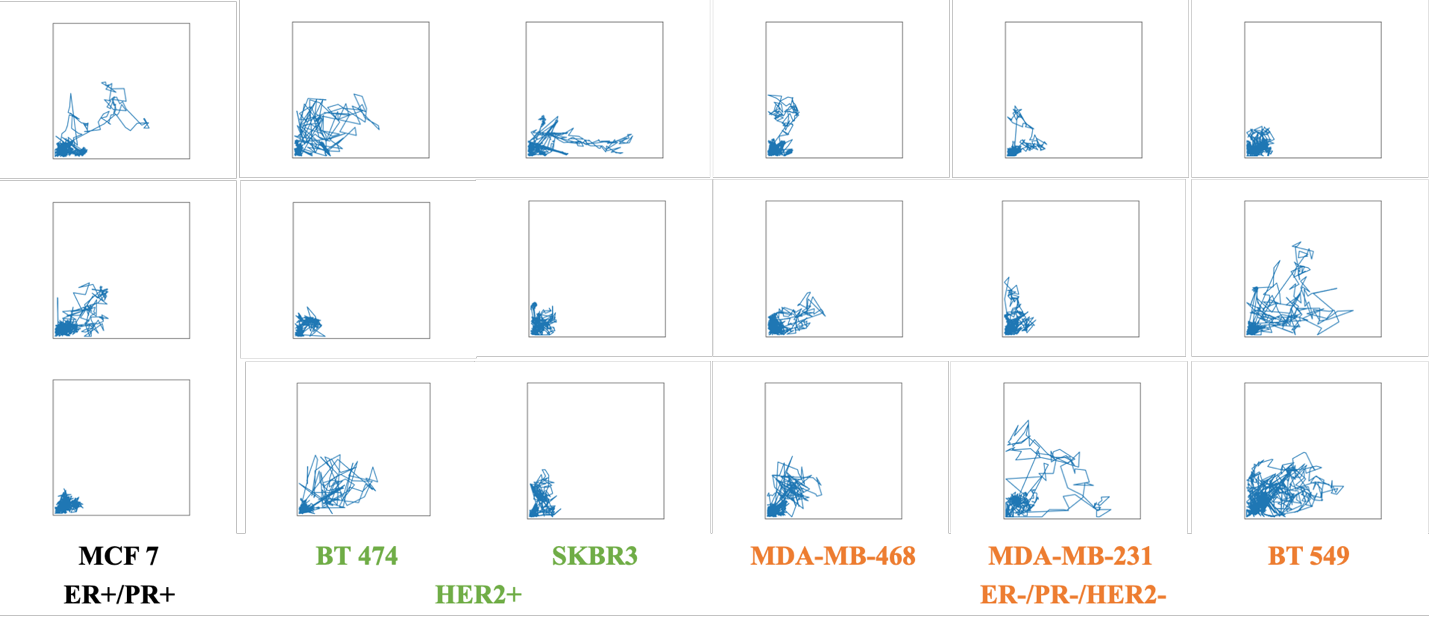


# FIGURE S2 | Example trajectories after preprocessing

The HR+ cell line (MCF7) is marked with black, the HER2+ cell lines (BT474 and SKBR3) are marked with green, and the TN cell lines (MDA-MB-468, MDA-MB-231, and BT549) are marked with orange. The trajectories were translated near the origin to preserve the shape, but not the location within the field of view.


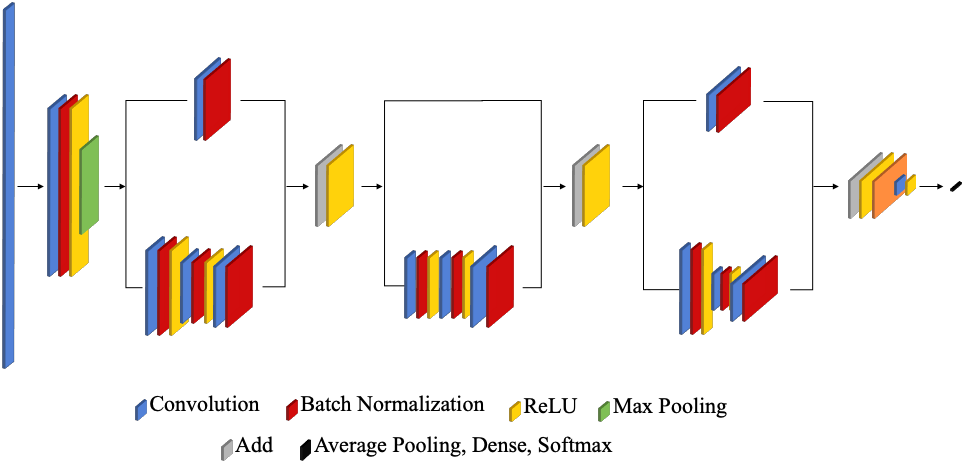


# FIGURE S3 | Model Architecture

Our model is based on a residual neural network (ResNet). The layers were organized in “blocks” with the convolution blocks having a single one-dimensional convolution layer in the skip connection and the identity block having a linear skip connection. Blocks were color-coded to represent different processes: blue: one-dimensional convolution layer, red: batch normalization layer, yellow: rectified linear unit (ReLU) layer, green: one-dimensional max-pooling layer, grey: add layer, black: one-dimensional average pooling, dense, a dropout layer and a softmax layer.

**
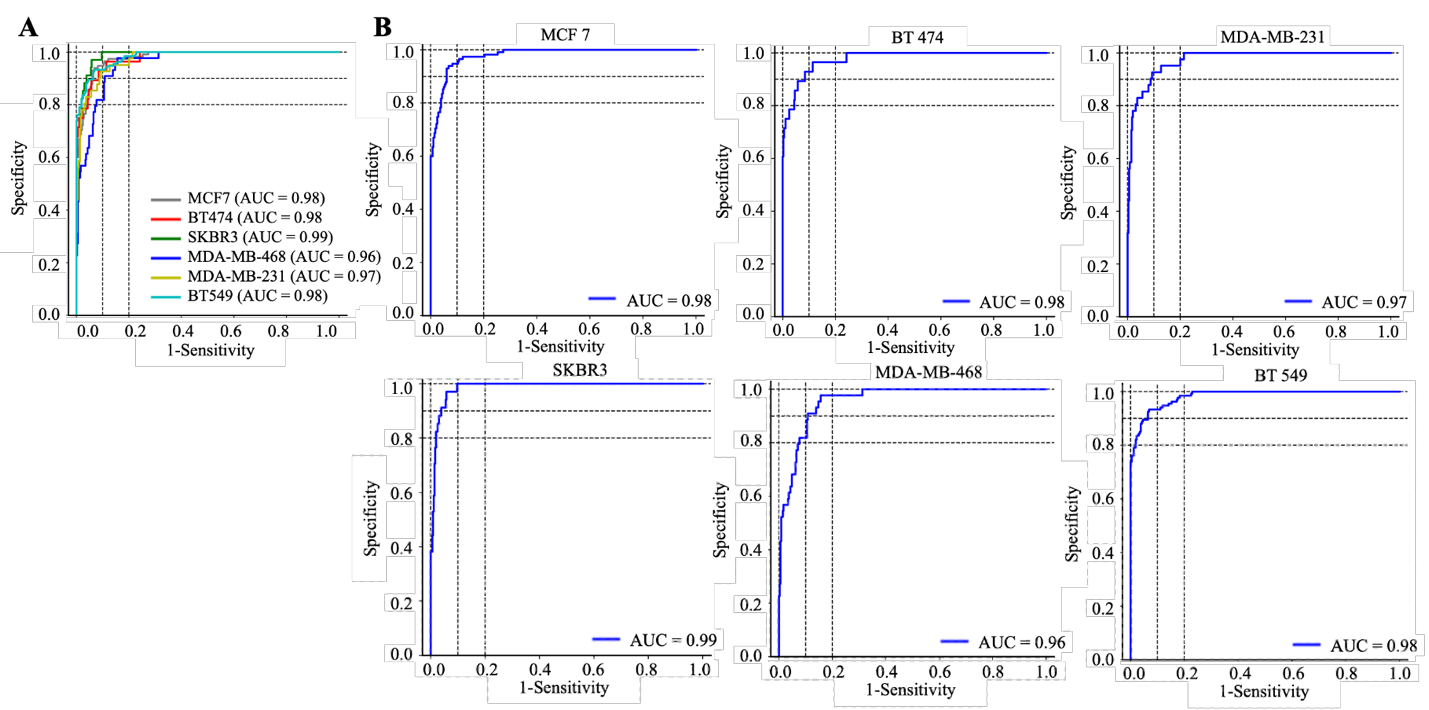
**

# FIGURE S4 | ROC curves of cell-line classification

**(A)** Composite of all the cell-line classification ROC curves with their AUC values (grey: MCF7, red: BT474, green: SKBR3, blue: MDA-MB-468, yellow: MDA-MB-231, cyan: BT549). The minimum AUC was achieved with MDA-MB-468 with 0.96, and the maximum AUC was 0.99 with SKBR3 classification. **(B)** Separate AUC curve for each cell line with the corresponding AUC values

**
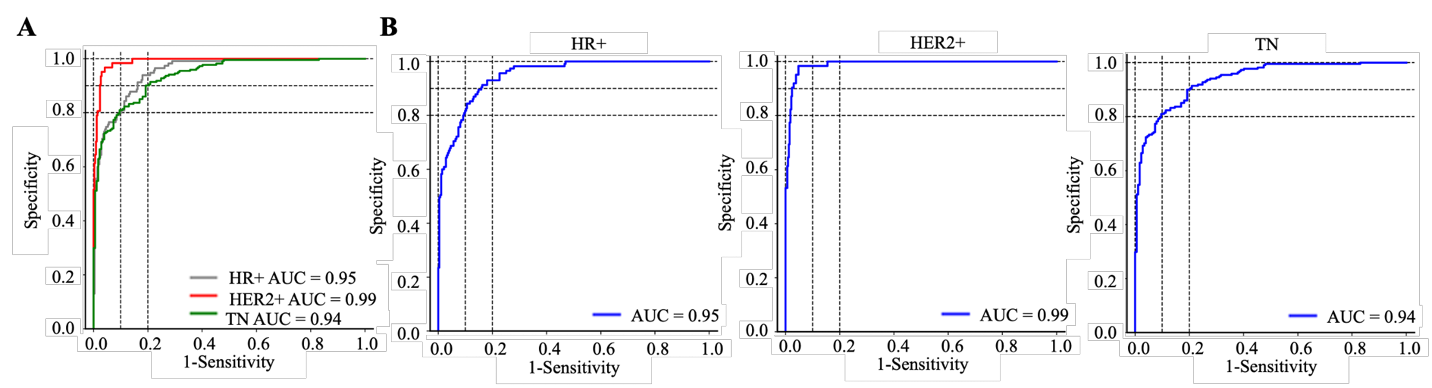
**

# FIGURE S5 | ROC curves of receptor status classification

**(A)** Composite of all receptor status ROC curves with their AUC values (grey: HR+, red: HER2+, green: TN). The HR+ classification achieved AUC of 0.95, the HER2+ classification achieved AUC of 0.99, and the TN classification achieved AUC of 0.95. **(B)** Separate AUC curves for each receptor status with the corresponding AUC values.


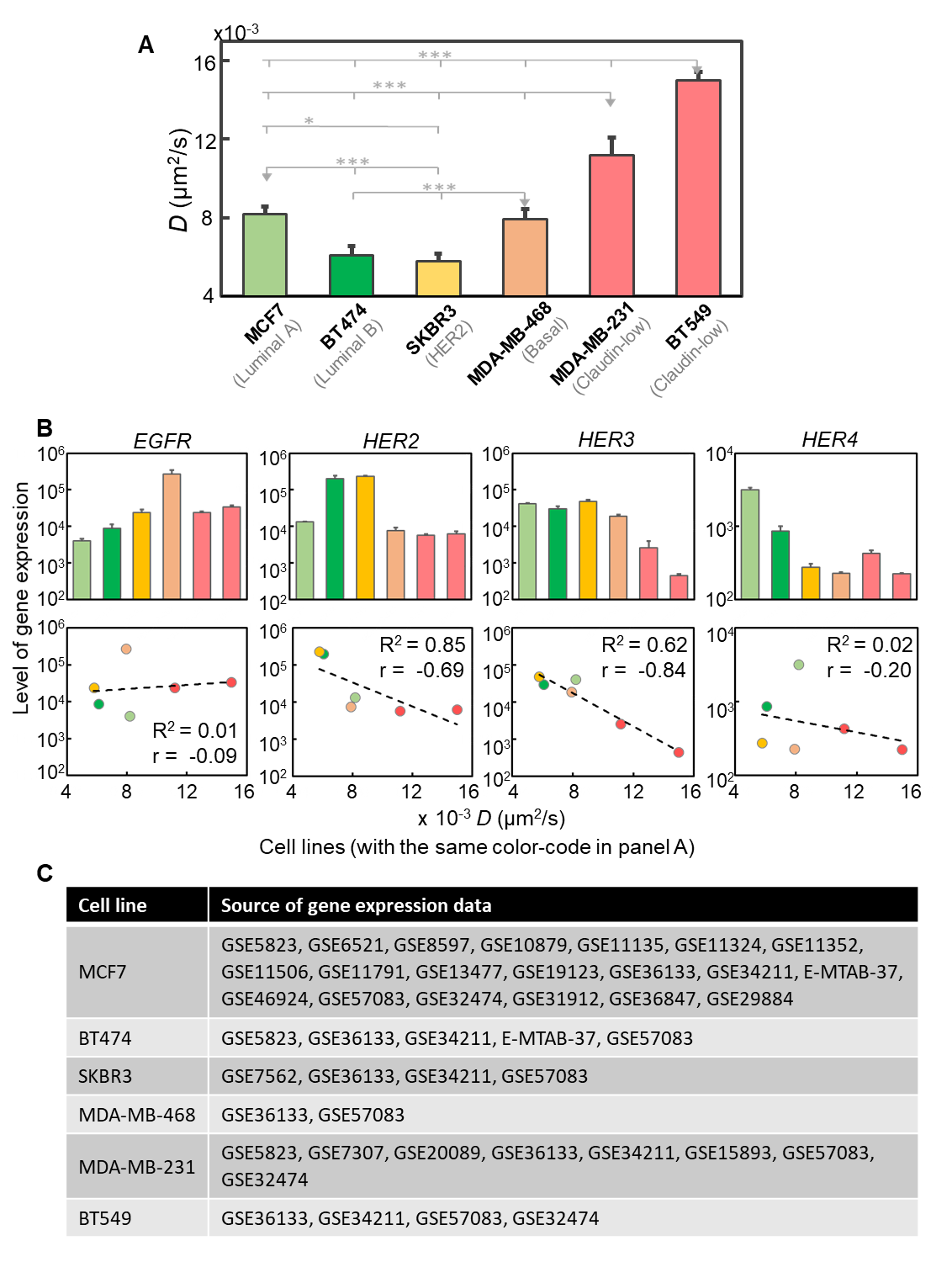


# FIGURE S6 | Correlations between EGFR diffusivity and expression levels of EGFR family

**(A)** The diffusivity of EGFR complexes measured in these six breast cell lines. The error bar represents the standard error. (**B**) mRNA expression levels of the EGFR family, including *EGFR*, *HER2*, *HER3*, and *HER4* among these cells lines. Each bar is color-coded with the same color corresponding to each type of cells shown in panel A. Correlations between expression levels of receptors to EGFR diffusivity. The R-squared is calculated by exponential curve fitting, and the r represents the correlation coefficient. (**C**) Source of gene expression data (mRNA microarray) analyzed on Genevestigator.


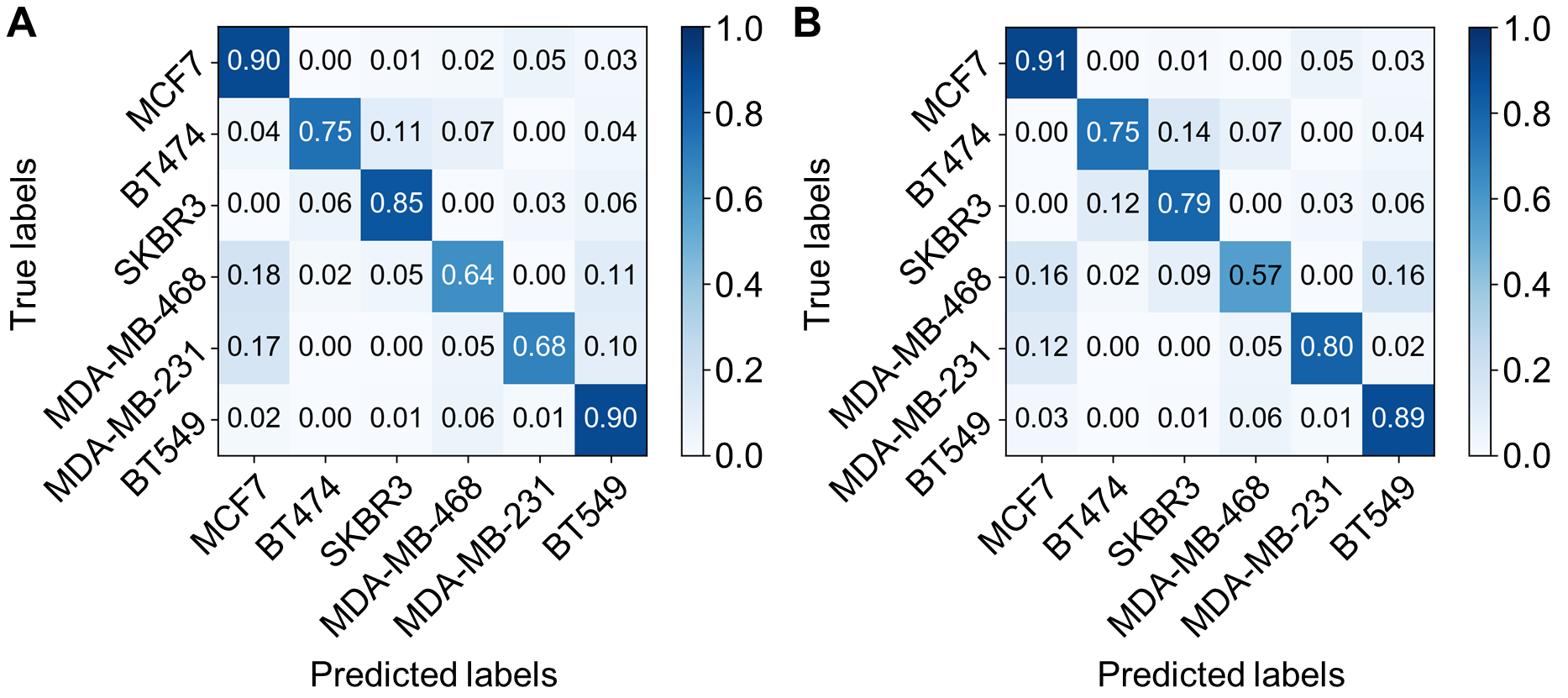


# FIGURE S7 | Cell line classification results with and without dropout layer

Normalized confusion matrix showing rates of correct classifications and misclassifications for each cell-line sample without dropout layer **(A)** and with a single dropout layer right before the final softmax layer **(B)**. An overall accuracy was slightly improved from 83.08% to 83.33%.


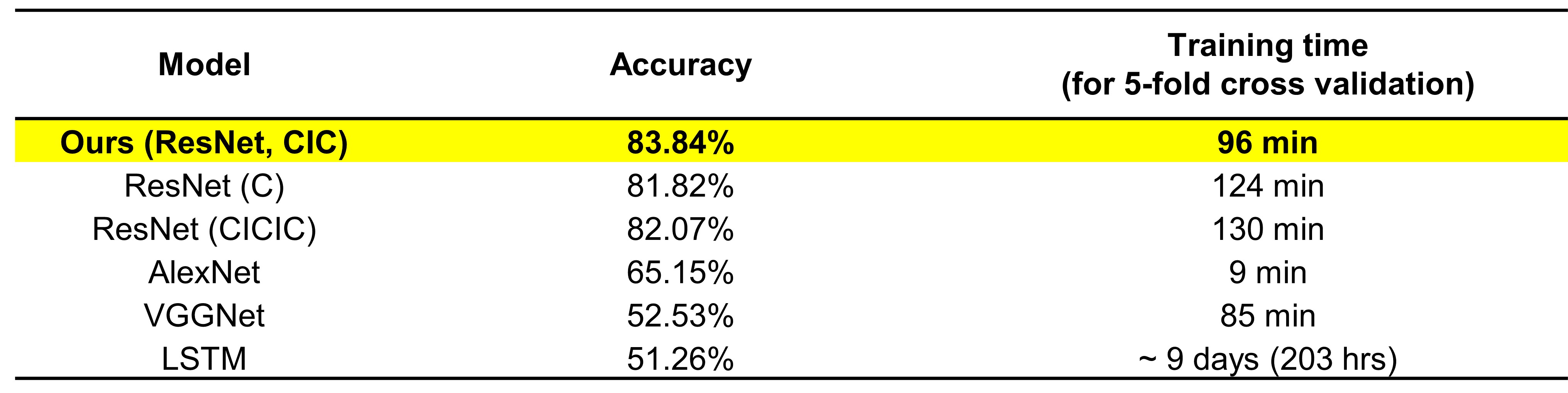


# TABLE S2 | Comparison results with different network architectures

Accuracy comparison with 5-fold cross-validation. Our network outperformed other networks in terms of overall accuracy. Due to the simple structures, AlexNet and VGGNet have shorter training time, but the overall performance was poor. LSTM, which is known as a suitable architecture for 1-D data, had the lowest accuracy and the longest training time.


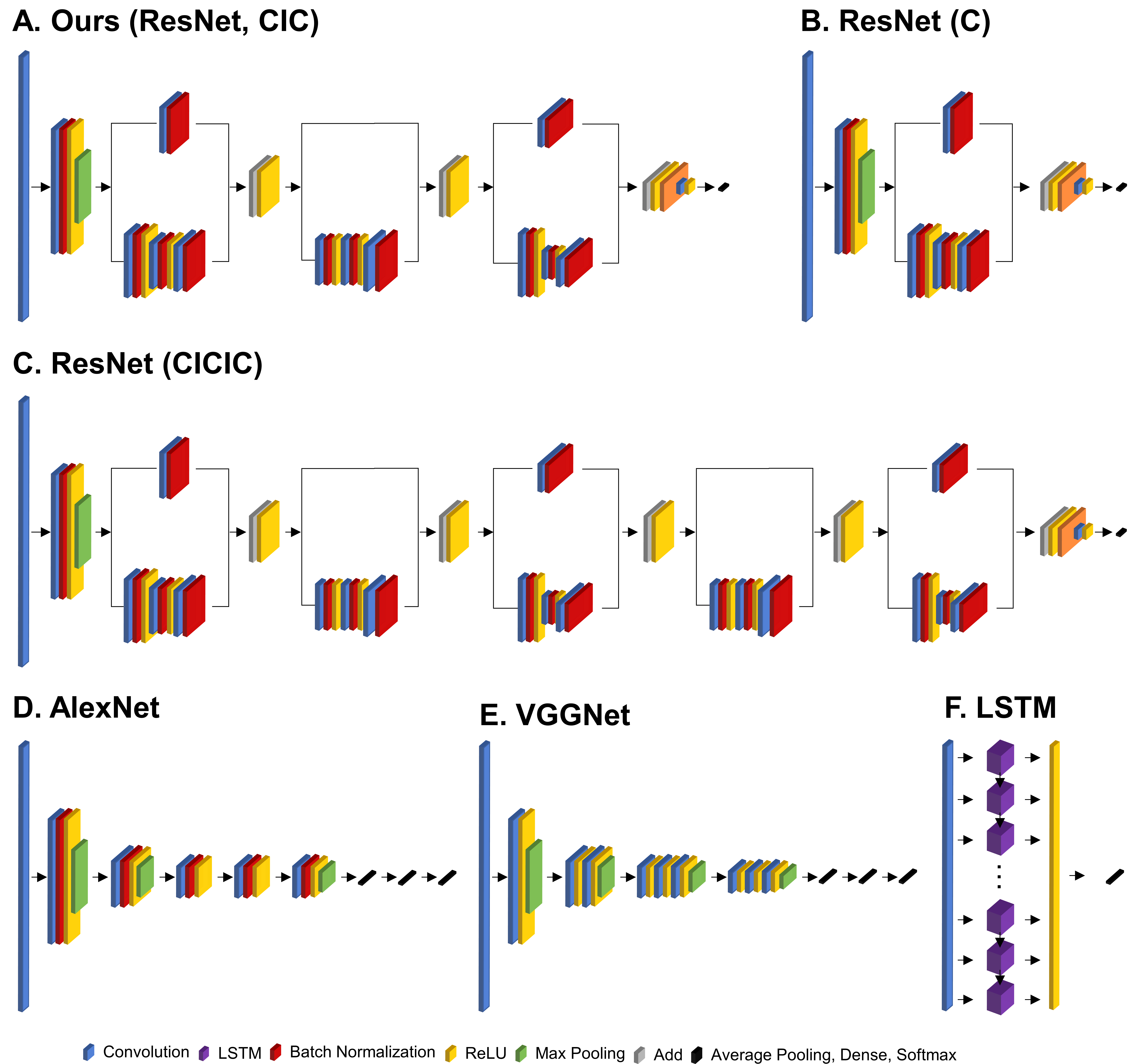


# FIGURE S8 | Model architectures for comparison results

(**A**) Our network consists of two convolution blocks with one identity block between them (CIC). We tested two different ResNet variants: (**B**) one convolution block and (**C**) three convolution blocks with two identity blocks (CICIC). Other than ResNet, (**D**) AlexNet and (**E**) VGGNet modified for the 1D dataset were employed. (**F**) Long short-term memory (LSTM) network was also compared.


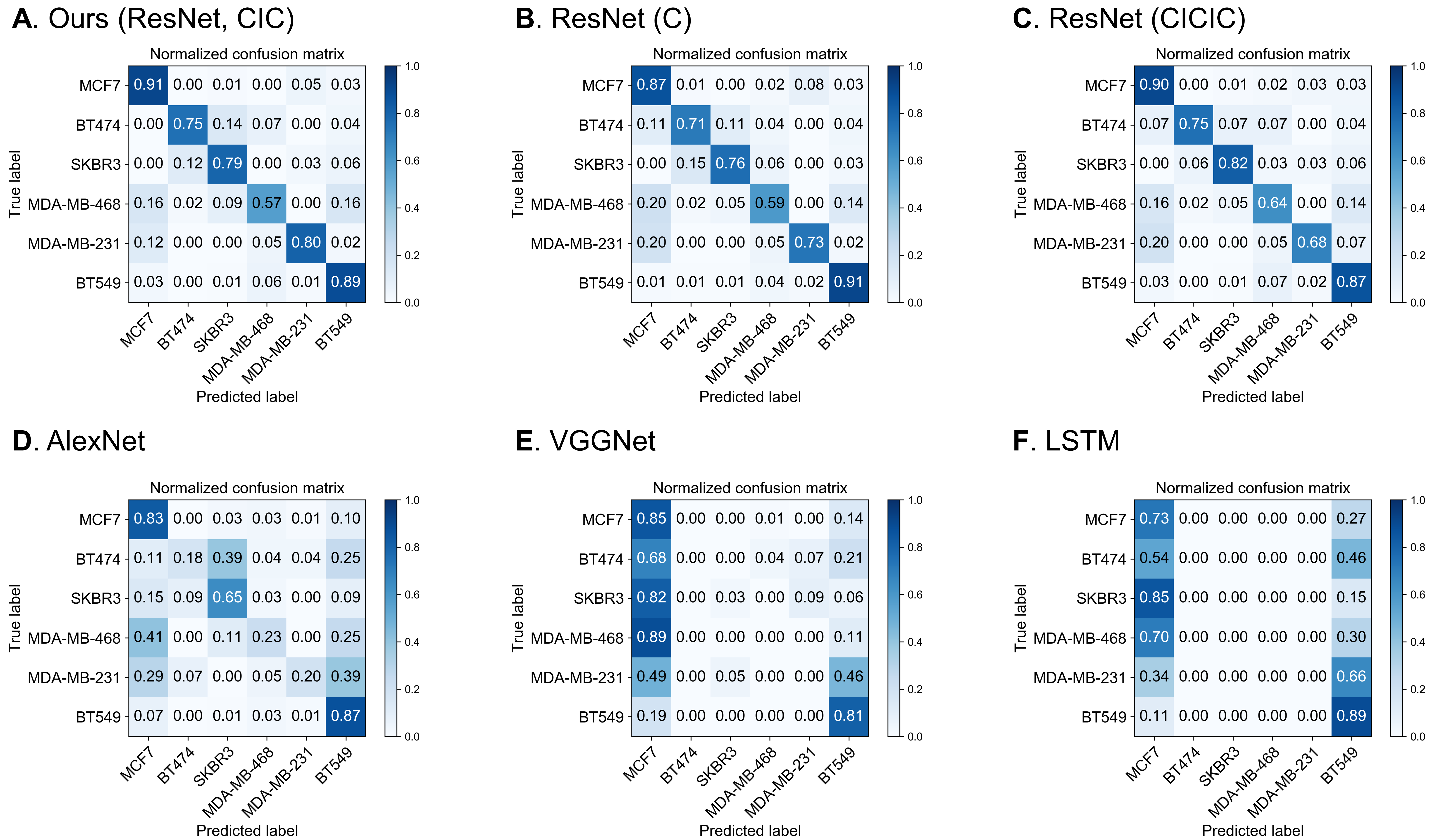


# FIGURE S9 | Confusion matrix with different model architectures.

Overall, (**A-C**) ResNet variant outperformed other networks based on (**D**) AlexNet, (**E**) VGGNet and (**F**) LSTM. (**A**) Ours with two convolutional blocks and one identity block (CIC structure) showed the best performance.

# Supporting References

Gers, F.A. and Schmidhuber, J. (2000) Recurrent nets that time and count. In, *Proceedings of the IEEE-INNS-ENNS International Joint Conference on Neural Networks. IJCNN 2000. Neural Computing: New Challenges and Perspectives for the New Millennium*. IEEE. p. 189-194.

Gers, F.A., Schraudolph, N.N. and Schmidhuber, J. (2002) Learning precise timing with LSTM recurrent networks. *Journal of machine learning research*;3(Aug):115-143.

He, K.*, et al.* (2016) Deep residual learning for image recognition. In, *Proceedings of the IEEE conference on computer vision and pattern recognition*. p. 770-778.

Kao, J.*, et al.* (2009) Molecular profiling of breast cancer cell lines defines relevant tumor models and provides a resource for cancer gene discovery. *PloS one*;4(7):e6146.

Krizhevsky, A., Sutskever, I. and Hinton, G.E. (2012) Imagenet classification with deep convolutional neural networks. *Advances in neural information processing systems*;25:1097-1105.

Li, X.*, et al.* (2019) Understanding the disharmony between dropout and batch normalization by variance shift. In, *Proceedings of the IEEE conference on computer vision and pattern recognition*. p. 2682-2690.

Liu, Y.-L.*, et al.* (2019) Assessing metastatic potential of breast cancer cells based on EGFR dynamics. *Scientific reports*;9(1):1-13.

McInnes, L., Healy, J. and Melville, J. (2018) Umap: Uniform manifold approximation and projection for dimension reduction. *arXiv*.

Neve, R.M.*, et al.* (2006) A collection of breast cancer cell lines for the study of functionally distinct cancer subtypes. *Cancer cell*;10(6):515-527.

Saxton, M.J. (1993) Lateral diffusion in an archipelago. Single-particle diffusion. *Biophysical journal*;64(6):1766-1780.

Simonyan, K. and Zisserman, A. (2014) Very deep convolutional networks for large-scale image recognition. *arXiv preprint arXiv:1409.1556*.

Subik, K.*, et al.* (2010) The expression patterns of ER, PR, HER2, CK5/6, EGFR, Ki-67 and AR by immunohistochemical analysis in breast cancer cell lines. *Breast cancer: basic and clinical research*;4:117822341000400004.

Thiery, J.P.*, et al.* (2009) Epithelial-mesenchymal transitions in development and disease. *cell*;139(5):871-890.

Yin, W.*, et al.* (2017) Comparative study of CNN and RNN for natural language processing. *arXiv preprint arXiv:1702.01923*.
